# “The show must go on”: How Paralympic athletes safeguarded their mental well-being and motivation to train for the postponed Tokyo 2020 games

**DOI:** 10.3389/fpsyg.2023.1099399

**Published:** 2023-03-30

**Authors:** Debbie Van Biesen, Sofie Morbee

**Affiliations:** ^1^Department of Rehabilitation Sciences, Faculty of Movement and Rehabilitation Sciences, KU Leuven, Leuven, Belgium; ^2^Virtus Academy, Virtus World Intellectual Impairment Sport, Sheffield, United Kingdom; ^3^Department of Developmental, Personality and Social Psychology, Ghent University, Ghent, Belgium

**Keywords:** motivation, COVID-19, basic psychological needs, selfregulation, performance, mental health, self-determination theory, para-sport

## Abstract

**Introduction:**

After the decision to postpone the Tokyo 2020 Games due to the COVID-19 pandemic, athletes had to adjust to a novel situation with feelings of uncertainty and insecurity. Grounded in Self-Determination Theory, this study was the first to examine whether different motivational profiles among Paralympic athletes can be identified, and to link these profiles with the athletes’ emotional, cognitive, and performance-related outcomes in times of a pandemic.

**Methods:**

Five months before the start of the Paralympic Games, the participants (*N* = 32; mean age = 33.2 ± 6.8 years) completed an online questionnaire measuring their demographics, basic psychological needs, perceived stress, depressive symptoms, general well-being, and motivational self-regulation strategies. Two months after the Games, they completed a second online questionnaire measuring their actual and perceived performance at the past Games.

**Results:**

Through K-means cluster analysis, three distinct clusters were identified based on the athletes’ dominant type of motivation, these are, dominantly amotivated (*n* = 11), autonomously motivated (*n* = 12), and controlled motivated (*n* = 9). Comparisons of athletes’ emotional, cognitive, and performance-related outcomes depending on their motivational profile revealed that the athletes with a dominantly amotivated profile had the least adaptive outcomes (i.e., low need satisfaction, high need frustration, and more depressive symptoms). Athletes with a dominantly autonomously motivated profile made less use of controlling self-motivating strategies compared to the other two profiles. Moreover, their actual performance at the Paralympic Games was better.

**Discussion:**

Although none of the athletes were at severe risk for depression or showed extremely high levels of stress, these results confirm that improving the quality of athletes’ motivation can safeguard their well-being and enhance performance in Paralympic Sports.

## Introduction

Across the world, corona measures were taken to contain the spreading of the COVID-19 virus (e.g., lockdown, wearing face masks, keeping social distance, etc.). Most, if not all, aspects of everyday life have been impacted by these measures, and the protracted nature of the situation has led to feelings of uncertainty and insecurity (Vermote et al., 2022), as well as negative mental health outcomes such as depression, anxiety, and traumatic stress in various cultures and populations (Boden et al., 2021). One of the hard-hit sectors was the sports sector (Asif and Toresdahl, 2022). In particular, athletes who were preparing for the Tokyo 2020 Paralympic Games were dealt a hard blow immediately after the first COVID-19 outbreak, since the decision to postpone the Games to the year 2021 was taken in March 2020 (IOC, 2020). This decision was followed by a year of uncertainty as to whether the Paralympic Games would actually take place because of the unpredictable nature of the pandemic, with its multiple waves. Moreover, the uncertainty remained until the last moment before departure (IPC, 2021), as the increasing number of positive cases in Japan during the precedent Olympic Games caused opposition among the local Japanese population. In this study, we examined whether the Paralympic athletes’ general motivation for partaking in their sport determined emotional, cognitive, and performance-related outcomes during the pandemic. More specifically, we considered several indicators of athletes’ well-being, their use of motivational self-regulating strategies to cope with the uncertain period leading up to the Paralympic Games, as well as their actual and perceived performance at the Games. The study was conducted among all Belgian Dutch-speaking Paralympians at two time points. The first assessment took place in March 2021, 1 year after the decision to postpone the Paralympic games, at the start of the third Corona wave in Belgium, and 5 months before the scheduled Paralympic Games in 2021. The second assessment was completed in November 2021, 2 months after the Paralympic Games took place.

### Emotional, cognitive, and performance-related impact of the COVID-19 pandemic

As suggested by the Self-Determination Theory (SDT) (Deci and Ryan, 2012), people are only able to become self-determined when their three basic psychological needs for autonomy, competence, and relatedness are fulfilled. First, a sense of autonomy occurs when people can make their own decisions, and feel in control of their own lives (Deci and Ryan, 2012). Second, the need for relatedness is fulfilled when people have meaningful social contacts (Patrick et al., 2007). Finally, the need for competence refers to the need of experiencing mastery of a task. In a recent study by Vermote et al. (2022) during the COVID-19 pandemic, it was demonstrated that the satisfaction of these three basic psychological needs related to better well-being, while need frustration predicted deteriorating effects on mental health over time. These and other authors (Šakan et al., 2020; Cantarero et al., 2021) have made a strong case for not seeing the satisfaction of the psychological needs for autonomy, competence, and relatedness as a ‘luxury good’, but considering the satisfaction of these needs important in times of insecurity, not at least to safeguard well-being. However, the COVID-19 measures caused a significant modification of people’s daily routines, thereby depriving or even undermining the three innate and universal psychological needs for autonomy, relatedness, and competence. In times of full or partial lockdown, the need for autonomy was under threat since many restrictions, postponements, or cancelations were made. For instance, closing down sports facilities like swimming pools disrupted athletes’ training routines. Even in countries where governments facilitated exceptions for professional athletes to continue their training practice, the international travel restrictions led to the cancelation or postponement of most competitive events. Likewise, athletes’ need for relatedness was under threat, as isolation and keeping distance (e.g., from friends, family members, and teammates) were core aspects of the measures imposed to prevent the COVID-19 virus to spread. Finally, especially for Paralympic athletes, the disruption of the preparation for one of the most important competitive events of their career potentially hampered or undermined their competence satisfaction (Puce et al., 2022). Moreover, given the postponement or cancelation of competitions, it was difficult for athletes to judge properly where they stood in terms of adjusting their intermediate goals compared to opponents.

All these stressors related to the COVID-19 pandemic came on top of the usual stressors experienced by Paralympic athletes in preparation for major competitions as previously identified by Jefferies et al. (2012) and Dehghansai et al. (2021), such as worries about their contribution to their team, the adequacy of their training and preparation, how they will manage psychosocial pressures, budgetary constraints, or injury prevention. Therefore, next to the Paralympians’ need-based experiences, we also considered their perceived stress and depressive symptoms. These are indicators of mental health which is an important indicator of well-being (Giles et al., 2020). There are reasons to believe that levels of depression and stress were increased among Paralympians during the COVID-19 pandemic, as they were in the global non-athletic (Xiong et al., 2020) and athletic (Di Fronso et al., 2022; Lambert et al., 2022) population. We know from studies on non-athletes with disabilities that the COVID-19 situation has had an even more negative impact on their mental health compared to the global population (Theis et al., 2021). Moreover, a critical scoping review of the literature on the impact of the pandemic on athletes with disabilities by Puce et al. (2022) identified 16 studies, 8 of which examined their mental health and/or well-being. A higher burden in para-athletes compared to athletes without disabilities was demonstrated, with more positive screenings for anxiety, depression, poor sleep quality (Nabhan et al., 2021), and a higher perceived negative impact on their training and performance of loneliness, psychological inflexibility, anxiety (Clemente-Suárez et al., 2020). On the other hand, Italian disabled athletes reported lower distress levels to adverse events compared to athletes without disabilities (Fiorilli et al., 2021) which was explained by the potential buffer effect of having the experience of living with impairment. Therefore, it remains unclear to what extent Paralympic athletes’ perceived stress and depressive symptoms were affected by the imposed COVID-19 restrictions.

In addition to possible effects on athletes’ well-being, the postponement of the Paralympic Games and the uncertainty about whether the Games would continue next year also implied a cognitive challenge, since it put pressure on athletes’ motivation to maintain their strict training schedule (Lambert et al., 2022). Paralympic athletes function at the highest level and therefore follow an extremely strict living and training schedule. Most athletes followed such schedules for at least 4 years to peak both mentally and physically at the Games, often with a rest period scheduled afterward. The postponement of the Paralympic Games meant that they had to maintain this strict schedule for another year, without being 100% sure that the Games would actually take place in 2021. Given this uncertainty, it was crucial for Paralympians to keep themselves motivated by employing cognitive motivational self-regulatory strategies. Motivational self-regulation involves the use of active coping strategies to modify or maintain one’s own motivation in difficult circumstances (Boekaerts, 1996; Engelschalk et al., 2016). These cognitive motivational self-regulating strategies can be more autonomous or more controlled in nature (Morbée et al., 2022). Autonomous self-motivating strategies aim at initiating the activity by arousing interest and reminding oneself of its relevance. Controlling self-motivating strategies, on the other hand, involve athletes’ strategies to initiate and persist in an activity by self-controlling their behavior. For instance, by reminding themselves that it is their responsibility to keep up their training schedule, by buttressing the successful completion of their training with feelings of pride and self-aggrandizement, by relying on external factors to get themselves going, or by projecting the controlling voices of others onto themselves. Previous research showed that autonomous self-regulation strategies are associated with less boredom, physical pain, and more life satisfaction, whereas controlled self-regulation strategies are associated with more boredom, physical pain, and reduced task pleasure (Waterschoot et al., 2021; Morbée et al., 2022).

But in the end, what elite-level athletes and their coaches are probably most interested in, is if and to what extent the postponement of the Paralympic Games affected the athletes’ performance. An additional year of training can be perceived as positive for the performance of athletes who were not yet at the top of their abilities when the final selection for the Games had to be made, providing them with more opportunities for additional training and growth. For athletes who leaned more toward the end of their careers, or were more vulnerable to injuries, an additional year of training in difficult circumstances might have had a deteriorating effect on their performance, with a lower chance of making the final selection or winning a medal. Previous research on the impact of the pandemic on athletic performance at the Games is scarce. The review by Puce et al. (2022) retrieved only one study that investigated the impact of COVID-19 confinement on performance outcomes in para-athletes (Schipman et al., 2022). Specifically, the authors recorded the results of the 10 best world performers in Olympic and Paralympic events since 2010, and noticed that the performance decrements were dramatical, as previously only observed during the two World Wars. The present study will add to the existing knowledge about the impact of the pandemic on performance, by including measures of actual and perceived performance.

However, although the aforementioned research showed that the pandemic most likely had emotional, cognitive, and even performance-related effects on Paralympic athletes, no study has examined what factors might explain why some athletes were more or less resilient during this uncertain but crucial period leading up to the Paralympic Games. Therefore, in this study, we examined whether Paralympic athletes’ motivation for their sport could determine whether they managed to respond more or less resiliently in terms of emotional, cognitive, and performance-related outcomes.

### Motivation to sport

According to SDT, one of the most influential contemporary motivational frameworks within sports psychology (Hagger and Chatzisarantis, 2007), both the quantity and quality of Paralympic athletes’ motivation play a key role in athletes’ emotional, cognitive, and performance-related sports experiences (Ryan and Deci, 2017; Bautista et al., 2019). Regarding the quality, three types of motivation can be distinguished, these are, autonomous motivation, controlled motivation, and amotivation. Autonomous motivation can be considered high-quality motivation because it entails experiencing a sports activity as self-initiated, enjoyable, or congruent with one’s interests and values (Vansteenkiste et al., 2010). Controlled motivation, on the other hand, involves the engagement in a sports activity based on external (e.g., reward, punishments) or internal (e.g., feelings of pride or guilt) pressured reasons, and thus represents a form of low-quality motivation (Vansteenkiste et al., 2010). Finally, amotivation reflects a total lack of intentionality. Previous research has convincingly shown that autonomous motivation and amotivation are associated with, respectively, the most and least desirable outcomes, such as athletes’ positive and negative affect, depressive feelings, and performance (Assor et al., 2009; Gillet et al., 2009; Haerens et al., 2018) whereas the correlates for controlled motivation fall in-between. However, the vast majority of these studies were carried out among low-competitive level athletes. Although laymen’s beliefs suggest that pressure may help professional athletes to push themselves beyond their limits and harden them to develop coping resources, empirical studies within elite athletes revealed that the different types of motivation yielded a similar pattern of correlates among professional athletes. To illustrate, autonomous motivation in elite athletes related to desirable outcomes such as doping avoidance and injury rehabilitation (Chan and Hagger, 2012; Chan et al., 2015), whereas controlled motivation and amotivation related to negative outcomes such as burn-out and symptoms of overtraining (Lonsdale and Hodge, 2011; Chan et al., 2015; Trčková and Burešová, 2019).

Regardless of their performance level, most athletes endorse multiple reasons to engage in sports which then get combined into specific motivational profiles, that is, a configuration of motives that provide greater insight into the overall motivational pattern of an athlete (Vansteenkiste and Mouratidis, 2016). There are only a few SDT-based studies that attempted to identify such motivational profiles in adult athletes (Gillet et al., 2009; Rottensteiner et al., 2015). Typically, four different profiles are distinguished (i.e., with athletes scoring high or low on both good and poor quality of motivation, or high on either and low on the other), each of which is differently associated with a diverse set of outcomes. However, only one of these studies has linked these motivational profiles to COVID-19-related outcomes (Morbée et al., 2022). The results of this study suggested that athletes with a more qualitative motivational profile (i.e., characterized by autonomous motivation to engage in sport) managed to handle the uncertain situation in more resilient ways compared to athletes with a poor qualitative motivational profile (characterized by high levels of controlled motivation and amotivation), or a high/low-quantity (characterized by, respectively, high/low levels of autonomous and controlled motivation). However, this study was conducted exclusively among non-disabled cyclists at all competition levels (from amateur to professional) and included only “soft” outcomes (i.e., basic psychological needs and motivational self-regulation strategies). Whether these findings can be generalized to disabled athletes at the highest international level, and whether motivation also plays a role in “hard” performance outcomes, remains unclear.

### Present study

It was the purpose of the present study to investigate emotional (i.e., basic psychological need satisfaction and frustration, stress, depressive symptoms, and general well-being), cognitive (i.e., motivational self-regulation strategies), and performance-related outcomes in relation to elite Paralympic athletes’ motivation while preparing for the Paralympic Games in a lockdown period. First, as a rather explorative aim, the scores on the emotional, cognitive, and performance-related outcomes were compared between athletes with various types of impairment (physical, visual, and intellectual impairment), gender (male versus female), type of sport (i.e., individual versus team athletes), and impact of COVID-19 on their training routines (more, less, or equal training volume during the pandemic compared to before). The first main aim was to identify the motivational profiles of Paralympic athletes based on the types of motivation as distinguished within SDT (i.e., autonomous motivation, controlled motivation, and amotivation). The second aim was to verify whether these motivational profiles were associated with several indicators of athletes’ well-being, their cognitive motivational self-regulation strategies to cope with the uncertainty that went along with the postponement of the Paralympic Games, as well as with their actual and perceived performance at the Paralympic Games. Based on the findings by Morbée et al. (2022), we expected Paralympic athletes with a profile characterized by high autonomous motivation and low controlled and amotivation to yield the most adaptive pattern of outcomes compared to athletes with a motivation profile dominated by controlled motivation or amotivation.

## Method

### Participants

The sample included all 32 (24 males and 8 females) Dutch-speaking Belgian athletes on the short-list to represent Team Belgium at the Paralympic Games in Tokyo, which took place in 2021. The age of the athletes ranged from 20 to 45 years (*M* = 33.22; SD = 6.75). The athletes competed in eight Paralympic disciplines, namely cycling (34%), boccia (3%), goalball (19%), athletics (16%), badminton (3%), para-equestrian (9%), wheelchair tennis (3%), and table tennis (13%). The majority of the participants competed in individual sports (81%) compared to 19% in goalball which is a team sport. The majority of athletes had a physical impairment (PI = 60%), followed by visual impairment (VI = 34%), and intellectual impairment (II = 6%). The self-reported impact of the COVID-19 pandemic on their training volume during the pandemic was neutral (i.e., no change in training volume) for 37.5% of the athletes, negative (less training volume) for 50% of the athletes, and positive (more training volume) for 12.5% of the athletes. Recruitment of athletes was facilitated by the disability sports confederation ‘Parantee-Psylos’ who approved this study and invited their athletes to participate. A summary of the demographic characteristics can be found in Table 1.

**Table 1:** Descriptive information about the participants.

Total (*n*)	32
Male/female ratio (*n*)	24/8
Age (M ± SD)	33.22 ± 6.75
**Sport (%)**	
Cycling	34
Goalball	19
Athletics	16
Table tennis	13
Para-equestrian	9
Wheelchair tennis	3
Badminton	3
**Impairment type (%)**	
Physical impairment	60
Visual impairment	34
Intellectual impairment	6
**Impact of Covid on training volume (%)**	
Neutral (no change)	37.5
Negative (less)	50
Positive (more)	12.5

### Procedure

**Figure 1:**
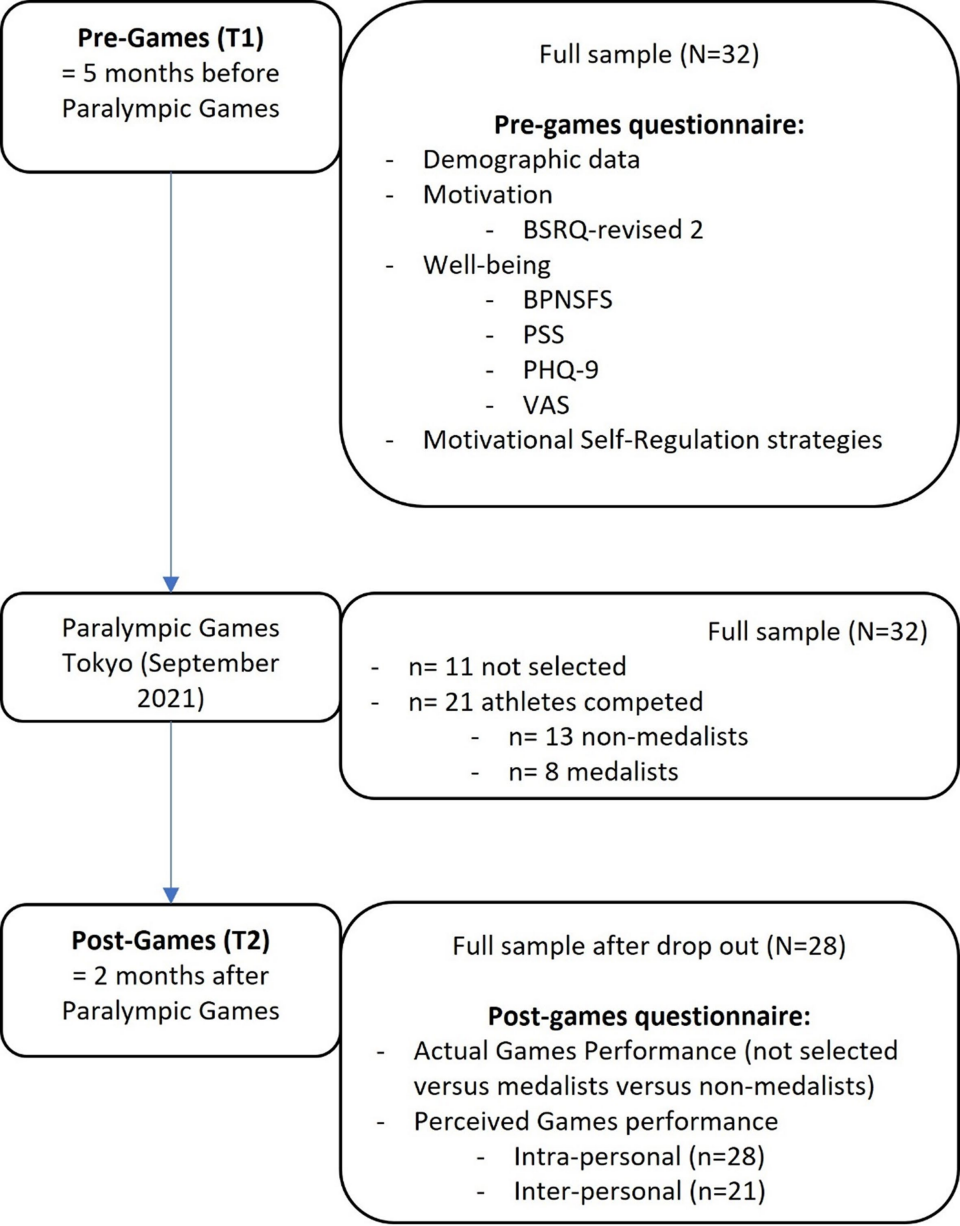
Flowchart of the procedure. BSRQ = behavioral regulation in sport questionnaire, BPNSFS = Basic Psychological Need Satisfaction and Frustration Scale, PSS = Perceived Stress Scale, PHQ = Patient Health Questionnaire, VAS = Visual Analog Scale.

An online survey format using the Qualtrics XM software was used for data collection at two moments in time. The first questionnaire was completed by the athletes in March 2021, 5 months before the start of the Paralympic Games, at the start of the third Corona wave in Flanders, in times of a partial lockdown. The second questionnaire was completed in November 2021, 2 months after the Paralympic Games took place. The first survey took approximately 25 min to complete, and the second survey took a maximum of 10 min. After having given their informed consent by e-mail, participants were given access to the online survey. The pre-games questionnaire consisted of participants’ demographic data (age, gender, type of sport, impairment classification, and training situation before and during the pandemic), several indicators of well-being, and their use of motivational self-regulating strategies. This first questionnaire was given to all Dutch-speaking Paralympic athletes on the short-list of Team Belgium and was filled out by 100% of them. The post-games questionnaire assessed the athletes’ actual and perceived performance during the Games. From the original sample of 32 athletes, four athletes dropped out for the second questionnaire because of mental health issues (*n* = 1), dissatisfaction with the non-selection for the Games (n = 2), or loss of interest in the study (*n* = 1). A visual representation (i.e., flowchart) of the procedure can be found in Figure 1. Ethical approval for the study was given by the Education-Support Committee (OBC) of KU Leuven.

### Materials

#### Pre-game questionnaire

##### Motivation

The shortened version of the Behavioral Regulation in Sport Questionnaire BSRQ-revised 2; adapted from Lonsdale et al. (2008), as successfully used in the study by Morbée et al. (2022) assessed the participants’ general sports motivation based on SDT. After the stem “I put effort in my sports…,” a total of 28 items surveyed three categories of autonomous motivation (16 items, e.g., “because I enjoy it”), controlled motivation (8 items, e.g., “because I would feel ashamed if I did not”), and amotivation (4 items, e.g., “but I actually wonder why”). The three scales were scored on a 5-point Likert scale from 1 (Totally not applicable to me) to 5 (Totally applicable to me). The internal consistencies in this study were good to excellent (α_autonomous_ = 0.84, α_controlled_ = 0.81, α_amotivation_ = 0.94).

##### Well-being

Basic Psychological Needs. A short version of The Basic Psychological Need Satisfaction and Frustration Scale (BPNSFS; adapted for sports from Chen et al. (2015)) was used for this study. The questionnaire has a total of 12 items with two items per need (autonomy frustration/satisfaction, relatedness frustration/satisfaction, and competence frustration/satisfaction), cumulated into the two variables need satisfaction (e.g., “I currently feel a sense of choice and freedom in the things I undertake for my sport”) and need frustration (e.g., “I currently feel insecure about my sporting abilities”). All items were scored on a 5-point Likert scale ranging from 1 (Totally not applicable to me) to 5 (Totally applicable to me). The internal consistencies in this study were acceptable (α_satisfaction_ = 0.69, α_frustration_ = 0.62).

Perceived Stress. The Perceived Stress Scale (PSS) is the most widely used instrument to assess self-perception of stress with established acceptable psychometric properties (Lee, 2012). By means of 10 items (e.g., “In the past month, how often did you feel that difficulties were piling up to the point that you could not overcome them?”) on a 5-point Likert scale from 0 (never) to 4 (very often), the degree to which individuals appraised situations as stressful during the previous month was assessed (Cohen et al., 1983). The cumulated score reflects the perceived stress on a scale from 0 to 40. The internal consistency in this study was good (*α* = 0.79).

Depressive Symptoms. The Patient Health Questionnaire (PHQ-9) is a validated diagnostic screening tool for the presence and severity of depressive symptoms (Gilbody et al., 2007; Nandakumar et al., 2019) during the past two weeks. The scale consists of 9 items (e.g., “little interest or pleasure in doing things?”) to be scored on a 4-point Likert scale ranging from 0 (not true at all) to 3 (almost every day). The internal consistency in this study was acceptable (*α* = 0.62).

General Wellbeing. A Visual Analog Scale (VAS) was added as a control tool in this study for the Paralympians to situate themselves on a continuum from 0 to 10 with respect to the general question of how they were feeling.

##### Motivational self-regulation strategies

The Motivational Self-Regulation Strategies in Sports Questionnaire (Morbée et al., 2022) assessed the Paralympians’ strategies to self-regulate their motivation. Based on 27 items, loading on autonomous motivational strategies (12 items; e.g., “finding out how the training can be personally valuable for me”), controlled strategies (12 items; e.g., “reminding myself that sometimes you have to do things in life against your will”), and lack of strategies (3 items; e.g., “I can not think of any ways to motivate myself to train”). Athletes completed this questionnaire on a scale ranging from 1 (Totally not applicable to me) to 5 (Totally applicable to me). The internal consistencies in this study were acceptable to good (α_autonomous_ = 0.73, α_controlled_ = 0.82, α_amotivation_ = 0.69).

#### Post-game questionnaire

##### Actual and perceived games performance

As an indicator of actual (i.e., objective) performance, the sample was divided into three subsamples based on whether or not they qualified for the final selection of athletes who effectively participated in the Tokyo Paralympic Games and whether or not they won a medal during the Games. From our sample, 11 athletes did not qualify for the final selection (i.e., non-selected athletes). Of the remaining 21 athletes who actually competed during the Games, 8 athletes brought one or more medals home (i.e., Paralympic medalists brought 10 medals in total) and 13 did not win a medal (i.e., Paralympic non-medalists).

To assess the perceived (i.e., subjective) intra- and interpersonal performance, the validated items from the questionnaire of Haerens et al. (2018) were used. However, the language was simplified to be understandable by Paralympic athletes with intellectual disabilities or brain injuries. The questionnaire consisted of two parts and was filled out by 28 of the athletes (21 athletes who competed during the Games and 7 non-selected athletes). For athletes who gave permission (*n* = 26), the same questionnaire was also filled out by their personal coach. In the first part, the intrapersonal perceived performance was assessed, defined as the extent to which the athletes (or coaches) were satisfied with the progression athletes had made during the preparation for the Games on a physical and technical level (i.e., body components), and on a tactical and mental level (i.e., the mind-components). The second part, measuring the interpersonal perceived performance, was only filled out by the 21 Paralympians (and their coaches) who participated in the Games. It assessed the general satisfaction with the athletes’ performance during the Games and how they judged this performance against competitors in the same category.

### Data analysis

In a series of preliminary analyzes, descriptive statistics were performed and the data set was checked for outliers. Spearman correlations were used to investigate the association between all study variables.

Before examining our main aims, the distribution across the athletes’ motivation scores, and their scores on the emotional, cognitive, and performance-related outcomes (using the non-parametric Kruskal Wallis H test) were compared between athletes with various types of impairment (physical, visual, and intellectual impairment), gender (male versus female), type of sport (i.e., individual versus team athletes), and impact of COVID-19 on their training routines (more, less or equal training volume).

The variables used for the cluster analysis were the standardized scores (z-value) for the participants’ general motivation. To detect motivational profiles, we performed a K-means cluster analysis, using the SPSS software (IBM SPSS Statistics 24, SPSS Inc., Chicago, United States) (Aim 1). Once the number of clusters (i.e., motivational profiles) was determined, a multivariate analysis of variance (MANOVA) determined the differences between the clusters in terms of the dependent variables (basic psychological need satisfaction and frustration, perceived stress, depressive symptoms, general wellbeing, motivational self-regulation strategies, and perceived performance). In addition, regarding the perceived performance, it was investigated whether the coach versus athletes judged the athletes’ performance differently using Repeated Measures Anova. Finally, a cross-tabulation was performed to analyze the differences between the motivational clusters in terms of actual performance (Aim 2).

## Results

### Preliminary analyzes

Descriptive statistics for all variables (mean values, SD, minimum, maximum, skewness, and kurtosis) can be found in Table 2. No cases were identified as outliers.

**Table 2:** Descriptive statistics for all study variables.

Domain	Variable	*N*	M ± SD	Min	Max	Skewness	Kurtosis
Demographics	Age	32	33.22 ± 6.75	20	±45	−0.50	−0.47
General motivation	Autonomous type	32	18.19 ± 4.38	11	27	0.02	−0.97
	Controlled type	32	66.19 ± 10.26	47	94	0.40	0.37
	Amotivation	32	5.63 ± 3.16	0	12	0.02	−0.96
Well-being	Need satisfaction	32	17.47 ± 2.98	9	24	−0.85	1.66
	Need frustration	32	5.63 ± 3.16	0	12	0.02	−0.96
	Depressive symptoms	32	2.16 ± 2.63	0	9	1.20	0.34
	Perceived stress	32	11.28 ± 5.31	3	21	0.14	−1.06
	General well-being	32	7.87 ± 1.41	5	10	−1.14	−0.96
Motivational self-regulation strategies	Autonomous strategy	32	20.84 ± 4.65	11	30	0.19	−0.21
	Controlled strategy	32	26.13 ± 8.66	8	43	0.07	−0.68
	Lack of strategies	32	1.75 ± 2.03	0	8	1.52	2.55
Perceived Performance (athlete-report)	Intrapersonal-body	27	10.48 ± 2.61	2	13	−2.23	5.55
	Intrapersonal-mind	28	10.43 ± 1.95	5	13	−0.98	0.67
	Interpersonal	18	8.67 ± 3.36	2	13	−0.40	−0.59
Perceived Performance (coach-report)	Intrapersonal-body	25	10.36 ± 2.18	5	14	−0.53	0.24
	Intrapersonal-mind	25	9.76 ± 1.71	5	14	−0.35	2.30
	Intrapersonal	18	10.33 ± 3.09	3	14	−1.03	0.66

The strength of the relation between all dependent variables based on Spearman rank correlations is shown in Table 3. Autonomous motivation was positively related to autonomous self-regulation strategies. Controlled motivation and amotivation were positively related to need frustration, depressive symptoms, controlled self-regulation strategies, and lack of self-regulation strategies, while being negatively related to need satisfaction. Moreover, controlled motivation was also negatively associated with general well-being, but positively related to autonomous self-regulation strategies.

**Table 3:** Spearman rank correlations between all study variables.

	Age	AM	CM	A	NS	NF	DS	PS	GWB	AS	CS	LS	PPARB	PPARM	PPARI	PPCRB	PPCRM	PPCRI
Age	1																	
Autonomous motivation (AM)	−0.01	1																
Controlled motivation (CM)	−0.08	0.08	1															
Amotivation (A)	−0.17	−0.54**	0.46**	1														
Need Satisfaction (NS)	0.25	0.13	.-0.45**	−0.46**	1													
Need Frustration (NF)	−0.27	−0.27	0.45**	0.51**	−0.41*	1												
Depressive symptoms (DS)	−0.27	−0.20	0.60*	0.43*	−0.25	0.42*	1											
Perceived stress (PS)	−0.24	−0.15	0.22	0.10	−0.03	0.28	0.35	1										
General well-being (GWB)	0.07	0.18	−0.49**	−0.33	−0.22	−0.20	−0.70**	−0.39*	1									
Autonomous strategy (AS)	−0.20	0.52**	0.38*	0.04	−0.07	0.12	0.17	0.16	−0.22	1								
Controlled strategy (CS)	−0.12	−0.15	0.37*	0.42*	−0.22	0.27	0.39*	0.05	0.01	0.13	1							
Lack of strategy (LS)	−0.11	−0.32	0.50**	0.40*	−0.11	0.30	0.58**	0.10	−0.28	−0.20	0.48**	1						
Intra body athlete (PPARB)	−0.22	0.25	−0.31	−0.12	−0.12	−0.11	−0.09	0.18	0.10	−0.10	−0.22	−0.10	1					
Intra mind athlete (PPARM)	−0.14	0.20	−0.17	0.24	−0.11	0.24	−0.07	0.13	0.22	−0.05	−0.10	−0.11	0.28	1				
Inter athlete (PPARI)	0.03	−0.07	−0.23	−0.14	0.18	−0.14	−0.39	−0.26	0.51*	−0.13	−0.18	−0.18	0.11	0.10	1			
Intra body coach (PPCRB)	−0.13	−0.14	−0.01	−0.14	0.21	−0.14	−0.32	−0.22	0.17	0.09	−0.18	−0.12	0.44*	−0.29	0.60*	1		
Intra mind coach (PPCRM)	−0.25	−0.08	0.07	0.22	−0.28	0.22	−0.05	−0.16	0.04	0.01	0.11	0.13	−0.08	−0.17	0.73**	0.40*	1	
Inter coach (PPCRI)	0.10	−0.32	−0.15	−0.35	0.26	−0.35	−0.41	−0.49*	0.45	−0.13	−0.06	−0.39	0.04	−0.18	0.87**	0.68**	0.67**	1

∗*p* ≤ 0.05 (two-tailed); ∗∗*p* ≤ 0.01 (two-tailed). PP = Perceived Performance, CR = coach report, AR = athlete report.

### Differences between groups based on impairment, type of sport, gender, and perceived impact of COVID-19

The athletes with visual impairments scored significantly lower on the use of autonomous self-regulation strategies (*M* = 18.09 ± 3.53) compared to athletes with physical impairments (*M* = 18.09 ± 3.53; *p* = 0.022), but did not significantly differ from the athletes with intellectual impairment (*M* = 19.00 ± 4.24).

Gender differences were found for some indicators of perceived performance with male athletes scoring higher compared to female athletes. These gender differences were found for both athlete- (*M*male = 9.85 ± 2.73 versus *M*female = 5.60 ± 3.05; *p* = 0.03) and coach-rated interpersonal performance (*M*male = 11.42 ± 2.39 versus *M*female = 8.17 ± 3.37; *p* = 0.04), as well as for the mind aspect of intra-personal performance rated by the coach (*M*male = 10.22 ± 1.48 versus *M*female = 8.57 ± 1.81; *p* = 0.03).

The athletes who engaged in an individual sport had significantly higher scores for autonomous motivation (M *=* 21.12 ± 4.71), compared to team athletes (M *=* 16.67 ± 3.27; *p* = 0.035).

The subsample of athletes who were positively affected by the COVID-19 pandemic in terms of training volume had a significantly higher score (*M* = 9.50 ± 0.58) on general well-being compared to the non-affected group (*M =* 7.92 ± 1.31; *p* = 0.04) and the negatively affected subsample (*M* = 7.40 ± 1.41; *p* = 0.007).

### Motivational profile (first Aim)

**Figure 2:**
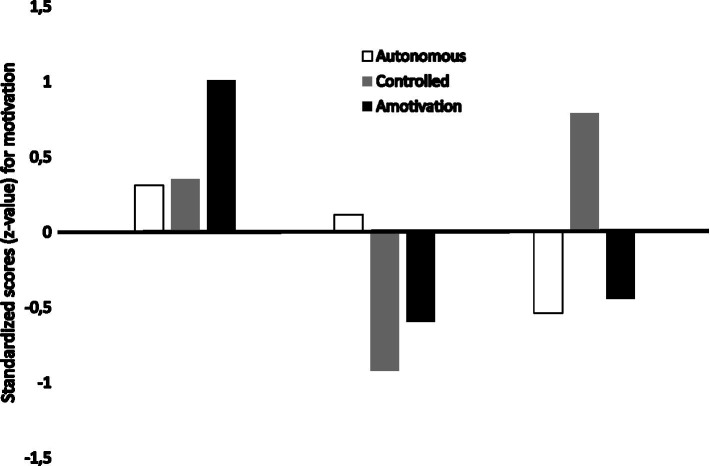
Visual representation of the three clusters of Paralympic athletes based on motivational profile.

Through K-means cluster analysis, three distinct clusters were identified based on the type of motivation of the athletes. Each cluster contains athletes with another dominant type of motivation. Figure 2 depicts the three clusters, labeled as dominantly amotivated (*n* = 11), dominantly autonomously motivated (*n* = 12), and dominantly controlled motivated (*n* = 9).

### Differences in emotional, cognitive, and performance-related outcomes depending on motivational profile (second aim)

The differences in athletes’ emotional, cognitive, and performance-related outcomes depending on their motivational profile are presented in Table 4. With respect to the basic psychological needs, athletes with a dominantly amotivated profile scored lower on need satisfaction compared to athletes with a dominantly controlled motivated profile and higher on need frustration compared to the other two profiles. They also reported more depressive symptoms and less general well-being compared to the dominantly autonomously motivated profile. Athletes with a dominantly autonomously motivated profile made less use of controlling strategies compared to the other two profiles. No other significant differences were found between the groups.

**Table 4:** Differences in well-being, motivational self-regulation strategies, and perceived performance between groups of athletes with distinct motivational profiles.

		Motivational profile (Mean ± SD)				
Domain	Variables	Dominantly amotivation	Dominantly autonomous	Dominantly controlled	*P*	post-hoc
Basic psychological needs	Need satisfaction	15.91 ± 3.21	17.33 ± 2.64	19.56 ± 1.94	0.019*	a < c
	Need frustration	8.82 ± 1.66	3.75 ± 2.98	4.22 ± 2.54	<001*	a > b = c
Well-being	Depressive symptoms	4.09 ± 2.70	0.75 ± 1.06	1.67 ± 2.63	0.004*	a > b
	Perceived stress	13.91 ± 5.63	11.00 ± 4.51	8.44 ± 4.77	0.066	
	General wellbeing	7.00 ± 1.16	8.42 ± 1.24	8.11 ± 1.54	0.047*	a < b
Motivational self-regulation strategies	Autonomous strategy	21.64 ± 4.13	18.42 ± 3.78	23.11 ± 5.23	0.052	
	Controlled strategy	29.55 ± 7.58	19.83 ± 7.06	30.33 ± 7.41	0.003*	a = c > b
	Lack of strategies	2.73 ± 2.69	1.17 ± 1.12	1.33 ± 1.80	0.141	
Perceived Perfomance (athlete-report)	Intrapersonal-body	10.10 ± 3.00	11.50 ± 1.43	9.5 ± 3.15	0.283	
	Intrapersonal-mind	10.5 ± 1.90	10.81 ± 1.25	9.7 ± 2.87	0.517	
	Interpersonal	7.33 ± 3.44	9.22 ± 3.19	9.67 ± ±4.16	0.511	
Perceived Perfomance (coach-report)	Intrapersonal-body	10.00 ± 1.33	10.40 ± 2.72	11.00 ± 2.65	0.719	
	Intrapersonal-mind	9.90 ± 1.29	9.50 ± 2.32	10.00 ± 1.22	0.833	
	Intrapersonal	9.00 ± 3.03	10.5 ± 3.06	13.50 ± 0.71	0.203	

*p < 0.05, SD = standard deviation.

**Figure 3:**
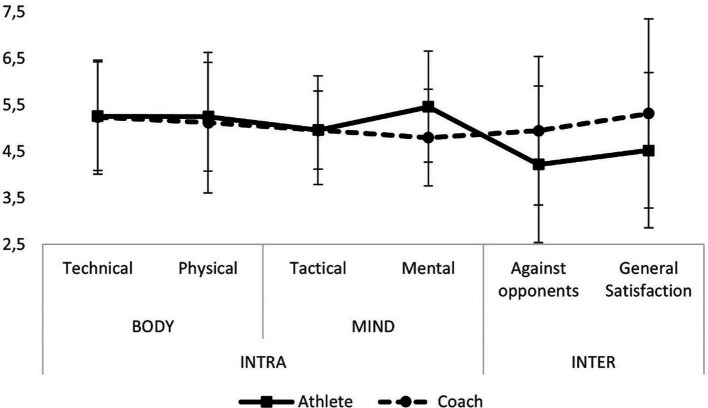
Comparison of perceived intra- and interpersonal performance between Paralympic athletes and their personal coaches.

In addition, regarding the perceived performance, we investigated whether the coach versus athletes judged the athletes’ performance differently. The results of perceived intra- and interpersonal performance by athletes and their coaches are shown in Figure 3. Intra-personal performance was not judged significantly different by athletes versus their coaches, apart from the mental aspect which was judged higher by athletes compared to the coach (*F* = 4.65, *p* < 0.05). The interpersonal performance was perceived as significantly higher by the coaches versus the athletes (*F* = 17.18, *p* < 0.001).

Finally, athletes’ actual performance depended upon their motivational profile (*χ*^2^ = 10.40, *p* = 0.03). None of the medal winners was predominantly amotivated, whereas 75% of them was predominantly autonomously motivated. Of the athletes that did not make the final selection, 45% was predominantly controlled motivated, 36% predominantly amotivated, and only 18% predominantly autonomously motivated.

## Discussion

One of the hard-hit sectors of the COVID-19 pandemic was the sports sector. Both recreational and elite athletes had to cope with uncertainty, regularly changing measures that impacted their training routines, and the cancelation of competitive events. Several studies indicated that the COVID-19 pandemic impacted athletes’ well-being and performance (e.g., Puce et al., 2022), but why some athletes suffered more than others remained unknown. Only one previous study looked at whether athletes’ ability to cope with the pandemic depended on their motivation (Morbée et al., 2022). The purpose of the current study was to shed new light on this topic by focussing on elite athletes with disabilities, thereby considering “hard” performance-related measures in addition to “soft” outcomes such as well-being and motivational self-regulating strategies.

### Wellbeing and athletic identity

An encouraging finding of our study was that Paralympians perceived their overall well-being positively during their preparation for the Tokyo Games, as indicated by two observations. First, 5 months before the Games, none of the athletes were at high risk for depression. The Patient Health Questionnaire is often used as a screening tool for diagnosing depression with a cut-off score of 10 (Manea et al., 2012) and none of the athletes in our sample obtained scores higher than nine. Our finding is in contrast with the study by Busch et al. (2022) who reported significantly lower scores on a short form of the PHS in German Paralympic athletes compared to a matched control group in the general population during eight measurement time points in the first year of the pandemic (March 2020 to April 2021). Second, a similar observation could be made regarding the perceived stress scale. None of the athletes in our sample showed alarmingly increased levels of stress, 12 of the athletes showed moderate stress levels (14–26 points), and the majority of athletes (*n* = 20) was situated in the low-stress category (0–13 points) (Lee, 2012). A similar finding was seen in the study by Fiorilli et al. (2021) who reported that only 8.22% of Italian athletes with disabilities compared to 30.14% of athletes without disabilities were affected by subjective distress during the pandemic. Although there is not a huge body of literature available about how the pandemic affects the perceived stress of athletes with disabilities (Puce et al., 2022, 2023) the opposite might have been expected because emerging research indicates that the COVID-19 pandemic increased psychological distress in the general population and even more among high-risk groups (Lorant et al., 2021). People with disabilities are seen as a high-risk group because they have less access to socioeconomic resources and supportive social networks (Goldmann and Galea, 2014). These and other unique stressors and challenges could worsen mental health for people with disabilities during the COVID-19 crisis. On the contrary, Paralympic athletes are a specific subgroup of people with disabilities, whose self-concepts are known to be tied to their athlete roles (Guerrero and Martin, 2018). Previous studies revealed that athletes with disabilities have equally strong athletic identities compared to athletes without disabilities (Groff and Zabriskie, 2006). A study that was performed on US Paralympians during the COVID-19 pandemic by Hu et al. (2021) looked at how athletic identity was affected by sport disruption. Most of the Paralympians in that qualitative study described their athletic identity as being challenged and negatively impacted. They experienced psychological struggles, mostly because of facility closures and cancelations of competitions. It was difficult for them to identify as athletes without the continuous training and high-level competition they practiced prior to the pandemic. The situation in Belgium might have been perceived more positively by the athletes in our sample, as the government facilitated exceptions for elite levels athletes (Olympians and Paralympians) to keep up with their training routines. Athletic identity was not measured in our study but we might assume that the Belgian Paralympians were able to identify with the athlete roles during the pandemic and safeguard their well-being as they were able to continue their physical preparations for the Games at least to some extent. Another possible explanation might be that elite Paralympic athletes have emerged as having better strategies to cope with stressful situations, because they are regularly facing these types of situations in their roles as athletes (e.g., performing under pressure in competition). Also, having an impairment might have brought them into several life experiences in which they have learned to adopt coping mechanisms (e.g., discrimination, inaccessibility) (Fiorilli et al., 2021). As such, they might have their adaptive cognitive emotion regulation skills better developed compared to others, which helps them to apply these strategies over the COVID-19 lockdown period.

### Characteristics of the motivational profiles

Previous research demonstrated that motivation is not a unidimensional construct and every athlete combines different types of motivation in a motivational profile (e.g., Vansteenkiste et al., 2009; Emm-Collison et al., 2020; Morbée et al., 2022). The first aim of this study was to identify the motivational profiles of Paralympic athletes based on the qualitatively different types of motivation as proposed by SDT (i.e., autonomous motivation, controlled motivation, and amotivation). Whereas Morbée et al. (2022) distinguished four motivational profiles in cyclists based on the quality and quantity of their motivation, the present study revealed only three clusters of motivational types in the sample of Paralympic athletes. The first profile was the one in which all types of motivation were present but with amotivation most dominant. The second profile was the one in which the autonomous type of motivation (good quality) was most dominant, although relatively low in quantity. In the third profile, the controlled type of motivation was the most dominant. The three motivational profiles were equally distributed among the athletes who were preparing for the Tokyo Games, with, respectively, 9 (dominantly amotivated), 11 (dominantly autonomously motivated), and 12 (dominantly controlled motivated) athletes matching the three profiles.

### Relation between motivational profile and emotional and cognitive outcomes

Beneficial outcomes (e.g., resilience to cope, need satisfaction, well-being) have been mostly attributed in the literature to the autonomous type of motivation whereas more negative outcomes (need frustration, ill-being) are more often related to amotivation or controlled motivation (Vansteenkiste et al., 2009; Standage and Ryan, 2020). Most apparent in our sample of Paralympic athletes was the detrimental effect of the predominantly amotivated profile on indicators of ill-being such as basic psychological need frustration, which corresponded to findings from previous studies (Morbée et al., 2022; Vermote et al., 2022). The athletes characterized by relatively high scores on amotivation scored lower on need satisfaction and higher on need frustration compared to the other athletes. Although the scores on the checklist for depressive symptoms were not clinically problematic for the predominantly amotivated Paralympians, they scored significantly worse compared to the Paralympians in the other groups. Moreover, their general well-being was significantly lower and their perceived stress level higher although not significant (*p* = 0.06). It has been repeatedly shown in the literature (Langan et al., 2016; Cuevas et al., 2018; Haraldsen et al., 2021) that a motivational profile characterized by a combination of controlled motivation and amotivation (i.e., “poor quality”) is the least adaptive of all profiles, and related to negative outcomes such as ill-being, anxiety, and perfectionism. In this study, we confirm that this relation is also upheld for elite athletes with disabilities.

With respect to the use of self-regulation strategies, the athletes characterized by predominantly controlled motivation and amotivation in our study were more likely to adopt more controlling self-regulatory strategies compared to the athletes with predominantly autonomously motivational profiles. On the other hand, we did not find proof in our study that the Paralympians with a predominantly autonomously motivated profile would also have more resilient responses to cope with the postponement of the Paralympic Games. This finding is in line with the results of Morbée et al. (2022), who also did not find a significant difference between the motivational profiles in terms of autonomous motivational self-regulation strategies. An explanation might be that the predominantly autonomously motivated profile was characterized dominantly by autonomous motivation in the absolute sense, although relatively low compared to the dominance of autonomous motivation in the dominantly amotivated profile. An alternative explanation might be that all participants already found resilient ways to cope with the pandemic given our survey was conducted after about a year of living with the COVID-19 pandemic.

### Performance

Whereas positive outcomes such as mental well-being and resilience have been attributed to good quality (i.e., predominantly autonomously motivated) motivational profiles in previous research, there is a lack of knowledge about the process and the mechanisms by which motivation affects performance in elite athletes. This study wanted to contribute to filling this gap in the literature, being one of the first in which the motivational profile of athletes is investigated in relation to a “hard” indicator such as performance in a highly competitive sports context. In the sports science literature (including disability sports science), a lot of attention has been dedicated to performance optimization by means of “hard” sciences such as sports physiology (e.g., effects of physiological parameters on goalball technical performance) (Alves et al., 2018) or biomechanics (e.g., optimal wheelchair configuration in para-sports) (Rietveld et al., 2021). It is more recently that researchers and practitioners started to apply concepts from “softer” sciences such as sports psychology for performance optimization. It might be due to the less tangible resources used in sports psychological interventions or unfamiliarity with this relatively novel area of research impeding athletes or researchers to implement it in their training practice (Gee, 2010). The most common use of sports psychology in relation to performance optimization is by means of the ‘negative’ approach, i.e., providing strategies to the athlete to cope with factors that potentially decrease their performance (e.g., anxiety, stress). There is only a paucity of studies aiming to unravel how positive factors such as good quality types of motivation enhance performance during competitions. In the majority of these studies, the findings revealed that autonomous motivation is associated with the best objective measures of performance, for example in youth tennis players (Cece et al., 2020), youth table tennis players (Martinent et al., 2018), judokas (Gillet et al., 2010), and female esthetic group gymnasts (Koka et al., 2020). We are not aware of studies examining the relation between athletes’ motivation and performance in elite Paralympic athletes. However, in an experimental study by Cheon et al. (2015), a coach intervention was implemented to help Paralympic coaches to adopt a more motivating (e.g., autonomy-supportive) style during the preparation for the 2012 London Paralympic Games. Results showed that athletes of coaches in the experimental condition were significantly more likely to win a medal than were athletes of the coaches in the control condition who applied a more demotivating (e.g., controlling) coaching style. Although not explicitly studied, better motivation of athletes with a more motivating coach could be at the root of their better performance. In our study, we obtained a similar finding, as Paralympic athletes’ actual performance at the Games was related to their motivational profile, with the majority of Paralympic medal winners (75%) belonging to the predominantly autonomously motivated profile whereas the majority of non-medal winners (54%) were predominantly amotivated and the majority of non-selected athletes (46%) identifying with the controlled motivational type. Future research is necessary but the results of our study are at least promising for future practice as it might help athletes and coaches to invest in the development of a good quality motivation for optimal performance. This contradicts the popular belief that coaches (especially at the highest levels) may do well to adopt a harsh, demotivating style with their athletes to help them reach their maximum potential (Jowett and Cockerill, 2003).

### Strengths and limitations

The main strength of this study is that it is unique in its kind, as we were able to recruit all the Dutch-speaking athletes on the Team Belgium Paralympic Games shortlist for the first questionnaire (response rate 100%), making it a fully representative sample. Moreover, the present study addressed a broad set of both “soft” and “hard” outcomes in relation to motivational profiles, including their actual and perceived performance at the Paralympic Games assessed in a multi-informant way.

However, some limitations of this study must be acknowledged when analyzing and interpreting the findings. First, the relatively small sample can be considered the main limitation. Therefore, the results of the cluster analysis should be interpreted with caution. Second, because of the purposeful sampling approach, we were not able to guarantee a balanced male–female ratio (male: 75%; female: 25%), nor a balanced ratio of impairment groups (PI: 60%; VI: 34%; II: 6%) or type of sports (individual: 81%; team: 19%). In future studies, we could increase the sample and the ratio by going beyond the borders and including Paralympic athletes from other nations. A third limitation concerns the reliability of the measuring instruments, with some scales of the questionnaires showing questionable internal consistency. Although the assessment tools that we used were proven valid and reliable in previous research, we might need to consider more adaptations to the instruments for use with athletes with intellectual, visual, and/or physical disabilities, including cerebral palsy. In an attempt to address this concern, we allowed the athletes with intellectual disabilities to have a trustee present to support them to fill out the questionnaire, but as a side effect, this might have reduced objectivity. Fourth, the type of design (cross-sectional) might also have influenced our results, since we assessed all variables (except for athlete performance) at one moment in time only. The motivation and well-being of a person can fluctuate from day to day, so the outcomes could be impacted by confounding variables we did not control (e.g., mood, the severity of COVID-19 restrictions). To reduce the influence of these confounding variables on internal validity, a follow-up test could have improved the accuracy of measurements in a test–retest design. The follow-up test we included in our own study only included measures of performance, but did not include variables measured at the first time point. We deliberately decided to limit the length of this follow-up questionnaire to avoid dropout. Another disadvantage of the cross-sectional designs is the inability to draw conclusions on causalities. Furthermore, the conceptualization and assessment of well-being in sports have been extensively debated by psychology scholars. There is a variety of definitions and various conceptual and theoretical perspectives on well-being (Giles et al., 2020). In our study, we used a combination of sport-specific measures of well-being (e.g., BPNSFS) and more general assessments (e.g., PHQ-9, PSS). A future study could benefit from a more conceptual approach taking into account the more recent views and available knowledge (Puce et al., 2023). Finally, we did not include a control sample of recreational or sub-elite athletes with disabilities or elite athletes without disabilities which might also have provided interesting comparisons.

### Future directions and practical implications

For future research, a longitudinal study design with a broader sample of athletes could be established to examine causal relationships and define specific characteristics within a motivational profile. Also, the differences in motivation and well-being of various types of Paralympic or Olympic athletes competing at different levels of performance, during times of a pandemic versus non-pandemic times could be considered. Based on the results of this study, we were able to draw some meaningful conclusions and recommendations for future support of Paralympic athletes toward coaches and support staff. The sports psychologist of the Paralympic team approved this study and is using the principles of SDT to support the athletes and coaches. Although the sporting world has now returned to a sense of normalcy, the threat of athletes testing positive for COVID-19 or any other infectious disease may be seen for years to come (Asif and Toresdahl, 2022). Therefore, in the future, it is recommended to teach the coaches how they can further support their athletes to adopt the most adaptive motivational profiles and apply self-regulation strategies to stay autonomously motivated in difficult circumstances. Furthermore, we recommend educating the athletes about their own motivational profiles, how they can enhance autonomous motivation, and the potential impact this might have on their well-being and performance.

## Conclusion

This study was unique in being the first of its kind to examine Paralympic athletes’ resilience in times of a global pandemic. An encouraging finding was that the well-being of Paralympic athletes was not significantly affected during their preparation for the Tokyo Games in difficult circumstances. We distinguished three motivational profiles, which were equally distributed among the sample (i.e., dominantly amotivated, *n* = 9; dominantly autonomously motivated, *n* = 11; and dominantly controlled motivated, *n* = 12). The Paralympians with a motivational profile that was characterized by predominantly amotivation to engage in sport managed to handle the uncertain situation evoked by the pandemic in less resilient ways. These athletes reported the highest level of need frustration and the lowest level of need satisfaction, and their well-being was lower compared to the other Paralympians with a profile characterized by high-quality motivation. Next, also Paralympic athletes’ actual performance during the Games appeared to be related to their motivational profile. Specifically, the majority of Paralympic medal winners (75%) belonged to the predominantly autonomously motivated profile, whereas the majority of non-medal winners (54%) were predominantly amotivated, and the majority of non-selected athletes (46%) identified with the controlled motivational type. The results of this study confirm the importance of appropriate psychological support for elite athletes, including those with disabilities. Improving the quality of their motivational profiles can safeguard their well-being and enhance performance in Paralympic Sport.

## Data availability statement

The raw data supporting the conclusions of this article will be made available by the authors, without undue reservation.

## Ethics statement

The studies involving human participants were reviewed and approved by KU Leuven/UZ Leuven. The patients/participants provided their written informed consent to participate in this study.

## Author contributions

DB conceptualizing and drafting the article, revising it critically for important intellectual content, final approval of the version to be published, and accountability for all aspects of the work. SM conceptualizing and revising the study critically for important intellectual content, final approval of the version to be published, and accountability for all aspects of the work. All authors contributed to the article and approved the submitted version.

## Conflict of interest

The authors declare that the research was conducted in the absence of any commercial or financial relationships that could be construed as a potential conflict of interest.

## Publisher’s note

All claims expressed in this article are solely those of the authors and do not necessarily represent those of their affiliated organizations, or those of the publisher, the editors and the reviewers. Any product that may be evaluated in this article, or claim that may be made by its manufacturer, is not guaranteed or endorsed by the publisher.
